# Pain Therapy Guided by Purpose and Perspective in Light of the Opioid Epidemic

**DOI:** 10.3389/fpsyt.2018.00119

**Published:** 2018-04-23

**Authors:** Amie L. Severino, Arash Shadfar, Joshua K. Hakimian, Oliver Crane, Ganeev Singh, Keith Heinzerling, Wendy M. Walwyn

**Affiliations:** ^1^Department of Psychiatry and Biobehavioral Sciences, David Geffen School of Medicine at the University of California Los Angeles, Los Angeles, CA, United States; ^2^Vatche and Tamar Manoukian Division of Digestive Diseases, Department of Medicine, David Geffen School of Medicine at the University of California Los Angeles, Los Angeles, CA, United States; ^3^Veteran Affairs Greater Los Angeles Healthcare System, Los Angeles, CA, United States; ^4^UCLA Brain Research Institute, Los Angeles, Los Angeles, CA, United States; ^5^Department of Psychiatry, Western University of Health Sciences, Pomona, CA, United States; ^6^Department of Family Medicine, David Geffen School of Medicine at the University of California, Los Angeles, CA, United States

**Keywords:** biased agonism, pharmacokinetics, opioid epidemic, chronic pain, opioid use disorder, oxycodone, mixed agonists

## Abstract

Prescription opioid misuse is an ongoing and escalating epidemic. Although these pharmacological agents are highly effective analgesics prescribed for different types of pain, opioids also induce euphoria, leading to increasing diversion and misuse. Opioid use and related mortalities have developed in spite of initial claims that OxyContin, one of the first opioids prescribed in the USA, was not addictive in the presence of pain. These claims allayed the fears of clinicians and contributed to an increase in the number of prescriptions, quantity of drugs manufactured, and the unforeseen diversion of these drugs for non-medical uses. Understanding the history of opioid drug development, the widespread marketing campaign for opioids, the immense financial incentive behind the treatment of pain, and vulnerable socioeconomic and physical demographics for opioid misuse give perspective on the current epidemic as an American-born problem that has expanded to global significance. In light of the current worldwide opioid epidemic, it is imperative that novel opioids are developed to treat pain without inducing the euphoria that fosters physical dependence and addiction. We describe insights from preclinical findings on the properties of opioid drugs that offer insights into improving abuse-deterrent formulations. One finding is that the ability of some agonists to activate one pathway over another, or agonist bias, can predict whether several novel opioid compounds bear promise in treating pain without causing reward among other off-target effects. In addition, we outline how the pharmacokinetic profile of each opioid contributes to their potential for misuse and discuss the emergence of mixed agonists as a promising pipeline of opioid-based analgesics. These insights from preclinical findings can be used to more effectively identify opioids that treat pain without causing physical dependence and subsequent opioid abuse.

## Oxycodone and Oxycontin at the Center of the Prescription Opioid Epidemic

### The History of Oxycodone Treatment of Chronic Non-Cancer Pain

Oxycodone, a semisynthetic opioid, was first formulated in 1916 from thebaine, a chemical found in opium poppy plants. The drug was first marketed as a less addictive alternative to “narcotic” drugs, such as morphine and heroin, which were typically prescribed to patients as an analgesic in the early 1900s. Oxycodone was first released in the USA in 1939 by Merck as a combination drug containing scopolamine, oxycodone, and ephedrine, but was discontinued in 1987 (1). Purdue Pharma then developed an extended-release formulation of oxycodone, called OxyContin. The FDA approved OxyContin in 1995 (2), noting that the reduced frequency of dosing was the only advantage of OxyContin over regular oxycodone (3). This drug was aggressively marketed by Purdue Pharma for opioid-based management of moderate-to-severe cancer and non-cancer pain where the use of an opioid analgesic was considered appropriate for more than a few days (2). Purdue used an aggressive marketing strategy to target-specific physicians (4), particularly those with less time to evaluate patients and often with less training in pain-management techniques. This led to more than half of the total OxyContin prescriptions being written by primary care physicians rather than pain specialists (4). In addition, direct-to-consumer pharmaceutical advertising, allowed only in the USA and New Zealand, has contributed to mass consumer awareness of the availability of these drugs with a demonstrated influence on the prescribing practice of physicians (5, 6). This aggressive physician directed marketing, as well as direct-to-consumer marketing, has become a benchmark for the marketing of opioids.

### The Financial Incentive for Prescription Opioid Distribution

The Purdue-Frederick company first marketed MsContin (morphine sulfate) as an extended-release opioid-containing formula to treat pain in terminal cancer patients. MsContin generated $475 million in sales over a decade. After the Sackler brothers acquired Purdue-Frederick and rebranded the company as Purdue Pharma, they released OxyContin, which generated $45 million in sales in just the first year after its release in 1996. By 2001, the annual revenue from OxyContin sales reached $1.1 billion and rose to $2.528 billion by 2014 in the USA alone.

Currently, the Purdue Pharma company is 100% owned by members of the Sackler family, who are worth $13 billion and ranked as the 19th wealthiest family in the USA in 2016 (7). In addition to Purdue Pharma and other Sackler holdings, there are several other companies manufacturing oxycodone and related opioid compounds to fill the 259 million annual prescriptions written to patients in the USA, generating an additional $11 billion in opioid sales annually in 2011 (8). These include Abbot Labs, Novartis, Teva, Pfizer, Endo Pharmaceuticals, Impax, Actavis, Sandoz, Janssen Pharmaceuticals, etc. Together, these figures demonstrate the significant financial incentive pharmaceutical companies have to market opioid compounds despite growing concerns of the abuse liability and safety of these drugs.

### Recognition of the Abuse Liability of Oxycodone and OxyContin

OxyContin was marketed as a delayed-release formulation allowing 12 h of continuous analgesia with fewer side effects than other opioid-based analgesics if used as directed. This formulation was promising in that the delayed-release would enable patients to sleep through the night, improving the standard of care for chronic pain patients at the time. However, this drug has been widely misused for non-medical purposes. At the time of the release of OxyContin in 1996, it was already known that 68% of an OxyContin tablet could be extracted by crushing the tablet (4). Since the first published reports of OxyContin abuse in 2000 (9), public awareness of its abuse liability has grown. Indeed, Purdue-Frederick, a holding of Purdue Pharma, paid $470 million dollars in fines to federal and state agencies and $130 million of payments in civil suits due to the misbranding of OxyContin as non-addictive in 2007 (10). Three executives of Purdue Pharma also pleaded guilty to OxyContin misbranding charges and paid $34.5 million in fines. By early 2017, there were daily reports of the diversion and misuse of prescription opioids with a number of states and counties across the country filing suit against five pharmaceutical companies, including Purdue. The plaintiffs in these suits claimed that the aggressive marketing campaign of opioid compounds is founded on fraudulent assertions of the safety of these drugs and that this misinformation has contributed to the ongoing opioid crisis. Purdue has responded to these claims by emphasizing that opioids are essential in pain management (2) and that their extended-release abuse-deterrent formulations are evidence of their drive to reduce the diversion of OxyContin (8). In 2018, Purdue stated it will no longer advertise directly to American doctors, a measure that will hopefully reduce over-prescription of opioids (11).

### The Patterns of Prescription Opioid Misuse and Overdose Mortalities Worldwide

The incidence of lifetime OxyContin abuse in the USA increased from 0.1% in 1999 to 0.4% in 2001 (12). By 2013, over 1,000 Americans were treated daily in emergency departments for prescription opioid misuse and in 2014, 4.3 million people used prescription opioids for non-medical reasons (13, 14). This trend was also seen in the number of deaths attributed to oxycodone, which increased from 14 cases in 1998 to ~14,000 cases in 2006 and 18,000 in 2015 (15). Although not of the same magnitude and somewhat delayed, this increase in opioid abuse and mortality is also occurring in other countries (16, 17). In Australia, oxycodone-related deaths increased sevenfold between 2001 and 2011 (18). In Finland, opioid mortalities increased from 9.5% of all drug overdose deaths in 2000 to 32.4% in 2008 (19), and data from Brazil, China, and the Middle East show similar increases in opioid diversion (17). In the United Kingdom, although tramadol and methadone are misused over oxycodone, the pattern of opioid misuse shows a similar increase to the USA albeit on a smaller scale (20). While Americans consume 80% of the global opioid supply and 99% of the global hydrocodone supply (21) and the number of overdose mortalities is considerably higher in the USA, the opioid epidemic is growing worldwide.

### The Most Vulnerable Populations

The incidence of opioid overdose mortality in the USA shows three hotspots: (1) the Appalachian states of Kentucky, Virginia, West Virginia, Pennsylvania, and Ohio, (2) the Northeast states of Maine, New Hampshire, and Rhode Island, and (3) the Southwest states of Nevada, Utah, New Mexico, and Arizona (15). This could be related to the demographics of these areas and the prescribing habits of the local medical professionals and pharmacies (22–25). Within all of these affected areas, opioid-related deaths are predominately Caucasians of middle age and are a result of drug overdose, alcohol-related disease, suicide, and psychiatric disorders. This has resulted in the first decline in life expectancy in the USA since 1993 (26–29). This has been highlighted in a series of articles that describe this population as subject to the “deaths of despair” (27, 30) and a “toxic stress” response to benign early-life events (31).

The primary factor contributing to these “deaths of despair” is the collapse of the white high-school educated working class from its heyday of the 1970s. This population’s struggles in the job market in early adulthood became more difficult over time and are accompanied by health and personal issues that contribute to an increased morbidity from chronic pain, and physical and mental health disorders including opioid use disorder [OUD (32)]. The (USA) National Bureau of Economic Research found that for every 1% increase in unemployment, there is a 3.6% increase in opioid-related deaths, suggesting that macroeconomic conditions have influence over national drug misuse (33). Considering the global economic aftershocks of the USA’s recession, we suggest that global economic recession contributed to the developing international opioid epidemic. To this point, a meta-analysis of research published from 1995 to 2015 in South America, the Caribbean, Europe, Asia, the USA, and Australia suggests that economic depression causes mental health issues that exacerbate illicit drug use (34). Case and Deaton additionally report that the use of prescription opioids did not create the vulnerable American profile, but the ease of availability of these compounds and the difficulty in treating opioid misuse in a depressed economy has inflamed the “sea of despair” that extends across the USA (27, 30).

### Addressing Chronic Pain in the Midst of the Opioid Epidemic

It is clear that mass production, marketing, and prescription of opioids for pain treatment has contributed to the opioid epidemic in vulnerable demographics, characterized by mental health disorders, socioeconomic challenges, and susceptibility to occupational injury. We discuss the interplay of mental health, pain, and depression, and how these factors contribute to the misuse and addiction of prescription opioids. One of the key marketing claims of pharmaceutical companies was that the presence of pain is protective against opioid misuse. The evidence for this claim is shockingly limited due to evolving diagnostic criterion for opioid misuse and does not account for the influence of mental health on opioid misuse behavior in the pain state. This gives us perspective toward treating pain with the intent to limit the pro-addiction properties and off-target effects of future pharmaceuticals to decrease opioid dependence in the chronic pain state. We look to insights from behavioral research on addiction and reward, and then to mechanistic research on the pharmacokinetic and signaling properties on opioids to address these issues.

## Are Chronic Pain Patients at Risk for Opioid Misuse?

### The Use of Opioids to Treat Chronic Pain

Opioids are highly effective analgesics for the management of acute pain or pain associated with cancer but it is the opioid treatment of non-cancer pain that is at the root of the opioid epidemic. Before the introduction of OxyContin, patients of all ages suffering from chronic non-cancer pain were commonly under-treated due to a fear of opioid addiction and of other side effects of these drugs (35–37). There were also few viable alternatives, heroin and its metabolite, morphine, had been abused during the Vietnam war and a prevailing public stigma against the use of drugs developed (38). This culminated in an “opioid-phobia” and recurrent under-treatment of pain. Spurred by the promise that the presence of pain protects against opioid addiction in patients with chronic cancer pain (39), the availability of a slow-release opioid (OxyContin) and an aggressive marketing strategy by Purdue, opioid-phobia was replaced by an over-willingness to prescribe opioids. This openness, based on the success of long-term opioid treatment of cancer patients by oncologists and pain specialists (39) was coupled with a lack of adequate physician training in the appropriate use of opioids or evidence for their use in other pain conditions, increasing market pressure and a lack of regulatory control by the government. This timely interplay of multiple factors resulted in the number of opioid prescriptions per 100 persons per year increasing from 61.9 in 2000 to 83.7 in 2009, and to 259 million prescriptions by 2012, almost one per person (40–42). This increase has not been reflected by a change in the percentage of either ambulatory Americans or those reporting to the emergency department in pain, suggesting that the increase in opioid prescriptions is unrelated to the presence or absence of pain (43, 44). However, these large scale epidemiological studies make it difficult to assess whether opioid-based treatment of the ~100 million Americans in pain (45) has influenced the incidence of opioid misuse that affects 4.3 million Americans (14, 46). Assessing the risk of opioid misuse in chronic pain patients provides greater insight into the vulnerability of these patients for addiction.

### The Risk of Opioid Misuse in Chronic Pain Patients

At the center of the opioid epidemic lies an unanswered question as to whether pain is protective of opioid misuse, a claim first made by Purdue in the 1990s. This was based on the findings from two studies that suggested the risk of addiction in pain patients was less than 1% (4). In the first study, Porter and Jick found iatrogenic addiction in 4 of 11,882 patients (47) and in the second, Perry and Heidrich found no addiction among 10,000 burn patients treated with opioids (48). A third study by Portenoy and Foley found no evidence of abuse behaviors in 38 patients treated with different opioids (49). The 5-sentence, 101-word letter to the New England Journal of Medicine in 1980 by Porter and Jick was recently found to have been uncritically cited by 439 authors as proof that addiction was rare in long-term opioid therapy. Despite its limitations, this letter and its citations have made a seminal contribution to the opioid crisis (50).

Before considering the evidence for a protective effect of pain in preventing opioid misuse, the criterion by which to assess opioid misuse must be defined. Initially, opioid dependence and addiction were considered the definitive benchmark. These terms have recently been replaced by the term “opioid misuse” or the use of opioids for any other reason or under any other dosing schedule than originally prescribed. The diagnostic classification system of patients misusing opioids has similarly evolved and the terms “abuse” or “dependence” have been replaced by the diagnosis of OUD. According to the criteria established by the Diagnostic and Standard Manual of Mental Disorders (DSM) V (51), OUD has levels of severity depending on the number of criteria met in several categories. The four categories of criteria that characterize OUD include impaired control, social impairment, risky use, and pharmacological properties (physical tolerance and withdrawal symptoms).

Using these criteria, recent reports clearly show that the incidence of opioid misuse and aberrant drug-related behavior is in fact higher, not lower, in pain patients compared with the general population (52–63). Chronic pain patients have a higher rate of comorbid depression and anxiety, likely contributing to their increased use and misuse of opioids (64). Indeed, 30–80% of chronic pain patients are concurrently diagnosed with both depression and chronic pain, a comorbidity known as the pain-depression dyad (65, 66). Both conditions are closely interwoven in that they respond to similar treatments, aggravate or improve each other, and share common biological mechanisms [for review see Ref. (67)]. Using opioids to relieve pain in the presence of this dyad may in itself drive further psychiatric comorbidities (68). This patient population is unsurprisingly more likely to increasingly misuse opioids (58, 63, 69–71). Patient escalation of opioid doses in response to the progressive interaction between pain and affect or to compensate for tolerance and changes in pain sensitivity over time (“pseudoaddictive” behaviors) (71–73) may explain enhanced aberrant drug-related behaviors in chronic pain patients (61), as well as the positive correlation between baseline pain and the presence of OUD at a 3-year follow-up (74).

## Why are Opioids so Addictive?

The motivation to continue taking drugs in spite of adverse consequences can be explained by several concurrent theories. The Opponent Process theory (75) results from a balance between two valuationally opposite components, a loss of function within the reward-mediating dopaminergic circuits and an increased function of stress-related circuitry involving the extended amygdala, the kappa/dynorphin opioid and corticotrophin-signaling systems [reviewed in Ref. (76)]. The latter system becomes hyperactive during opioid dependence and manifests as increased anxiety and aggressive behaviors (77, 78). Another, co-occurring theory of the motivation behind continued drug use is the Incentive Sensitization theory that proposes an increase in drug-paired cues with chronic drug taking (79). Together, these systems drive drug-seeking behavior that is a product of (1) a decrease in positive outcome coupled with the promise and pullof drug-associated cues and (2) an increase in dysphoria between drug exposures and during withdrawal (80–82). This is particularly relevant for opioids as these compounds induce a tolerance to repeated exposures of the same dose of the drug. This leads to (1) an escalating intake of opioids over time resulting in compulsive opioid-taking behaviors (83), (2) increasing dependence, and (3) increasing negative affect seen in the absence of the drug (84) that together culminate in further dysregulation of the reward system (85).

The negative affective state of depression and anxiety associated with chronic pain can be relieved temporarily by the analgesic and euphoric properties of acute opioid use, which contributes to their abuse liability in the chronic pain state (86). However, both pain and opioid use create a new homeostasis in the reward and stress-related pathways [reviewed in Ref. (87)], an example of which can be seen in chronic pain patients who misuse opioids and also fail to show a positive affect from natural rewards (84, 88). Preclinical studies in rodent models have been able to examine the interaction between pain and opioids at several levels. Pain does not affect the number of low doses of opioid infusions (of heroin, morphine, and oxycodone) earned in a self-administration model of drug-seeking behavior but does increase heroin self-administration to binge levels at higher doses and during prolonged access to the drug (89–93). By contrast, pain reduces the self-administration of fentanyl (94), a shorter-acting but highly efficacious opioid that rapidly crosses the blood–brain barrier (BBB) (95). Pain also increases drug (morphine)-seeking behavior when the drug is no longer available (96). This result suggests that the abuse liability of opioids in the chronic pain state is not directly motivated by analgesia-seeking and intensifies when the drug is no longer available yet drug-associated cues and environmental stimuli are present. Together, these preclinical findings suggests that chronic pain produces a vulnerability to addiction-like behavior, bearing a similarity to the behavior of opioid addicts in chronic pain who are more likely to relapse once tapering off a maintenance buprenorphine naloxone treatment (97).

## The Current Clinical Treatment of Chronic Pain Patients with Opioid Use Disorder

The current clinical treatment of chronic pain in patients with OUD in the USA relies on 3 FDA-approved medications: buprenorphine naloxone, methadone, and long-acting injectable naltrexone (98). These strategies seek to antagonize or minimize the agonist properties of opioids to reduce the likability of opioids. The use of methadone in the USA for OUD is limited to highly restricted methadone programs, but buprenorphine can be prescribed for office-based treatment by certified physicians. Buprenorphine, an opioid partial agonist, has analgesic effects and can be used to treat co-occurring chronic pain and OUD. While outcomes for OUD treatment with buprenorphine are similar for patients with and without chronic pain (99), poorly controlled pain during buprenorphine treatment is a risk for opioid relapse (97, 100, 101). Buprenorphine combined with naloxone, an opioid antagonist added to reduce diversion of buprenorphine for intravenous abuse, is FDA approved for OUD (e.g., Suboxone^®^), while a transdermal formulation (Butrans^®^) and a buccal film (Belbuca^®^), both without added naloxone, are approved for chronic pain. There are several novel compounds and approaches under development to treat pain, non-opioid compounds such as those that target cannabinoid receptors (102) and non-pharmaceutical, behavioral-based options to treat pain patients (103). However, for patients with chronic pain who continue to prefer a “quick fix from pain pills,” the development of analgesic compounds that are not rewarding and have minimal off-target effects remains a challenge in the current context of the opioid epidemic.

## Not All Opioid Analgesics are the Same: Exploring Novel Pharmaceutical Approaches to Guide Therapeutic Interventions for Chronic Pain

Opioids have been used for centuries as the treatment of choice for pain but “abuse-deterrent” formulations may decrease opioid misuse and deaths if strategically developed. Abuse-deterrent formulations of existing opioids are one strategy to reduce misuse, but they have been demonstrated to be modifiable, necessitating the consideration of additional properties to minimize abuse liability and fatalities. We suggest that therapeutics that do not produce reward are most likely to reduce diversion for misuse. Focusing on this approach, we discuss novel interventions that maximize analgesic properties while minimizing reward-promoting effects on the affective state. To provide background information for this section, we have included a table (Table 1) of the clinical use and pharmacological properties of opioids commonly used in the clinic and those that are often abused. This table shows that most opioids used clinically to relieve pain are either full or partial agonists of the mu opioid receptor (MOR) with some activity at other members of the family of opioid receptors.

**Table 1:** Descriptive and clinically relevant information of common opioids including clinical formulations, class of opioid, clinical uses, and cellular targets.

Drug [brand or alternative name(s)]	Common clinical formulation(s) (USA unless stated otherwise)	Classification; origin	Clinical use	Cellular target
Buprenorphine (e.g., Suboxone, Subutex, Buprenex)	Buprenorphine hydrochloride, buprenorphine naloxone (4:1)	Semisynthetic opiate; thebaine of the opium poppy (104)	Analgesia and maintenance therapy or opiate addiction treatment (Step 3 pain medication) (104)	Partial MOR agonist, KOR antagonist, nociceptin receptor agonist and antagonist (105, 106)

Fentanyl (e.g., Actiq, Duragesic, Fentora)	Fentanyl citrate	Synthetic opioid; *N*-phenethyl-piperidone (95)	Chronic and acute pain; administered orally, IV, transdermal patches (Step 3 pain medication) (107, 108)	Full MOR agonist, weak KOR agonist (109)

Heroin (i.e., diamorphine)	Diamorphine (UK) (110), diacetylmorphine (Canada/Switzerland) (111)	Opiate; morphine, and opium poppy (112)	Strong analgesic (Step 3 pain medication) (113, 114), opiate addiction treatment (Switzerland, Netherlands, Germany, England, Denmark) (115)	Partial MOR agonist (116) acts as prodrug (see active metabolites).

Hydrocodone (i.e., dihydrocodeinone) (e.g., Zohydro ER, Vicodin)	Hydrocodone bitartrate, hydrocodone bitartrate, and acetaminophen (117)	Semisynthetic opioid (118, 119); codeine (from opium poppy)	Chronic pain and opioid maintenance therapy (117)	Full MOR agonist (118)

Hydromorphone (e.g., Dilaudid)	Hydromorphone hydrochloride (120)	A semisynthetic opioid; the hydrogenated ketone of morphine (121)	Acute and chronic analgesia (Step 3 pain medication) (122), 5–8× more potent than morphine (123)	Full MOR agonist, partial DOR agonist, and weak KOR agonist (124, 125)

Methadone (e.g., Dolophine)	Methadone hydrochloride [(R) or racemic mixture] (126, 127)	Synthetic opioid (128); diphenylacetonitrile (129); and 1-dimethylamino-2-chloropropane (130)	Opioid dependence treatment (detoxification), chronic pain (131, 132)	Levo: full MOR agonist (109); dextro (d) NMDA antagonist (127).

Morphine (e.g., morphine sulfate ER, Roxanol, MsContin)	Morphine sulfate	Opiate; opium poppy (133)	Acute and chronic pain (Step 3 pain medication) (134)	Partial MOR agonist, weak DOR agonist (109, 135)

Oxycodone (e.g., Oxycontin, Norco, etc.)	Oxycodone hydrochloride, oxycodone acetaminophen, and oxycodone aspirin	Semisynthetic opiate; thebaine of the (136) opium poppy (137, 138)	Acute and chronic pain; may be superior than morphine for some types of pain (Step 3 pain medication) (139, 140)	Medium MOR agonist, partial KOR agonist (141), and partial DOR agonist (137, 142)

Remifentanil (e.g., Ultiva)	Remifentanil hydrochloride (always administered IV)	Synthetic opioid (143); derivative of fentanyl (144)	Acute pain or sedation (50–100× more potent than morphine) often used for surgical procedures (145–148)	Full MOR agonist (143)

Tramadol (e.g., Ultram)	Tramadol hydrochloride [racemic (+/−)], Tramadol hydrochloride, and acetaminophen	Synthetic opioid; salicylic acid with addition of 3-methoxyphenyl magnesium halide (149)	Moderate pain (Step 2 pain medication) (149, 150). Analgesic potency is 10% that of morphine (149)	(+/−) MOR agonist (151), (−) monoamine reuptake inhibitor (152)

*Alternative names refer to either the chemical name (referred to as i.e.) or brand name (referred as e.g.). Pain medication steps of analgesia are derived from World Health Organization classifications*.

*MOR; mu opioid receptor, DOR; delta opioid receptor, KOR; kappa opioid receptor*.

### Biased Agonism of the Mu Opioid Receptor

Over the years, many opioid compounds have been classified by their efficacy to activate a downstream pathway (such as cAMP), their selectivity for a specific opioid receptor, and ability to desensitize, internalize, and re-sensitize the ligand-bound receptor. More recently, many opioids have been further classified by their ability to induce a specific ligand-receptor conformation to recruit and activate different downstream signaling cascades [reviewed in Ref. (153)]. This bias toward either activation of G-protein-dependent or G-protein-independent, arrestin signaling pathways is known as “biased agonism” (154). This is an exciting discovery with obvious translational significance if specific pathways can indeed be activated to reduce non-analgesic opioid signaling. For MORs and other G-protein coupled receptors, such as the Cannabinoid 1 receptor, agonists biased toward arrestin-mediated signaling rather than G-protein-dependent signaling pathways seem to produce greater adverse side effects (155, 156). This has led to an emphasis on developing compounds that do not recruit either of the non-visual arrestin isoforms very well. Herkinorin was the first example of a MOR agonist showing that reduction of arrestin-signaling bias is associated with reduced adverse side effects. Using this logic, novel MOR agonists such as TRV130, TRV0109101, and PZM21, have been developed that do not recruit arrestin very well and also induce fewer adverse side effects (156–159). In particular, TRV130 has been shown to be G-protein biased, has a greater or equal analgesic potency to morphine, and induces less tolerance (160, 161). However, it is controversial as to whether TRV130 causes less rewarding behaviors, inhibition of gastrointestinal transit, or induction of respiratory depression than morphine (158, 160, 161). This compound is now in a Phase III clinical trial for parenteral treatment of acute pain (NCT02656875). TRV0109101 is also biased toward G-protein signaling and does not induce hyperalgesia, a common side effect of chronic opioid use (159). PZM21 similarly does not recruit β-arrestin 2 but is less potent than morphine and appears to induce less constipation, less respiratory depression, and reduced reward-seeking behaviors (158).

Using agonist bias profiles to predict the abuse of the commonly abused semisynthetic and synthetic opioids yields mixed results. For example, morphine shows the same or greater arrestin bias than oxycodone (162, 163), yet oxycodone has a greater abuse liability than morphine (136). Fentanyl and its analogs are highly abused yet this class shows no overt bias for either signaling pathway. This suggests that biased agonism alone cannot be used to separate the analgesic from rewarding properties of opioids.

### Pharmacokinetics

The action of opioids in the central nervous system facilitates analgesia mediated at supraspinal sites, such as those in the rostral ventral medulla, but also induces euphoria due to signaling at different central opioid receptor populations mediating reward (164). These central effects of opioids are also the major cause of overdose lethality due to respiratory depression (165), which is mediated by opioid receptors in breath-pattern generating neurons such as those in the pre-Bötzinger’s complex of the medulla (166). Limiting the access of opioids to the central nervous system is a beneficial pharmacokinetic manipulation that may bypass these off-target effects while preserving the potential for analgesia mediated by signaling at opioid receptors in the spinal cord or primary nociceptive afferent neurons.

This relationship between the pharmacokinetic profile of opioids and their abuse liability was first described in the 1970s (167, 168) and resulted in the use of buprenorphine and methadone as a non-rewarding analgesic or to treat OUD (104, 167, 169). It is now well-known that the intrinsic abuse liability of an opioid is a product of different pharmacokinetic parameters such as the time to peak plasma concentration, lipid solubility, BBB transport (a combination of passive diffusion and active transport in and out of the brain), and the presence of bioactive metabolites. Abuse liability may also be influenced by availability, with some compounds such as remifentanil being less available than others, such as methadone and heroin. We have outlined these pharmacokinetic properties and the abuse potential of commonly abused opioids and those used clinically (Table 2). This shows that fentanyl is one of the most rapidly bio-available opioids but has the same elimination half-life as morphine. However, it is highly lipid soluble (580× that of morphine) and so more easily crosses the BBB in both directions, shortening its effective duration of action. Heroin is a prodrug that is quickly transported across the BBB and converted to 6-acetyl-morphine, morphine, and demethylated to hydromorphone (170). Both of these opioids have a high abuse liability, but fentanyl and its derivatives are both more potent and have a longer elimination half-life making the fentanyl family of opioids fatal if taken in unknown or high quantities, as has often been the case (171). In comparison, morphine is hydrophilic, has poor protein binding capacity and its transport across the BBB is regulated, making it less likely to be abused. Compared with morphine, oxycodone is actively transported across the BBB, has a more rapid onset of effect and several active metabolites that all contribute to its greater abuse profile. At the other end of the spectrum are methadone and buprenorphine with medium-to-low abuse liabilities explained by low BBB permeability and a longer elimination half-life, in addition to differences in receptor selectivity and pharmacological profiles (Tables 1 and 2).

**Table 2:** The abuse liability, aspects of the pharmacokinetic profile, and bioavailability of select clinical and abused opioid compounds.

Drug	Abuse liability	Onset of effect and time to peak plasma concentration (min to h)	Elimination half-life (generally oral/human)	Metabolite(s)	Metabolite half-life	Bioavailability and blood–brain barrier (BBB) permeability/transport
Buprenorphine	Low in relation to other opioids (172, 173)	Sublingual onset of 0.25–0.75 h, peak plasma concentration at 2 h (174)	3–48 h (175), variable	Buprenorphine-3-glucuronide, norbuprenorphine-3-glucuronide (106)	Unknown (106)	28–51% bioavailability (176), low BBB permeability (177)

Fentanyl	Very high (178–180)	2–5 min onset of action, and peak plasma concentrations of 20 min after oral and 12 min after intranasal administration (95)	1.5–7 h (181)	Norfentanyl; minimal activity (182)	N/A	50–90%, highly lipophilic and high BBB permeability through passive and active transport (178, 183). Transfer half-life of 4.7–6.6 min (95)

Heroin	Very high (184)	45 s to onset of effect, heroin undetectable in blood and CSF by 20 min in rats (185)	3 min (IV) (170)	6-Monoacetylmorphine (6-MAM), morphine, and morphine’s metabolites (182)	6-MAM < 10 min after BBB crossing (116). Plasma conversion to morphine: 1.5–4.5 h, hydromorphone: 5 h, M6G: 2 h, M3G: 1.5 h	High (lipophilic) 60% or greater BBB permeability (116, 186)

Hydrocodone	High (136, 179, 187)	10 min to onset of effect and peak effects within 30–60 min (188)	3–9 h (189)	Hydromorphone and norhydrocodone (190).	Hydromorphone: 5 norhydrocodone: 8 (191)	25% bioavailability; 50% BBB permeability (187)

Hydromorphone	High (179)	5–30 min to onset of action, 30 min to peak effect (125, 189)	2–3 h (192)	Hydromorphone-3-glucuronide (182)	1.5–3 h (193)	55% bioavailability (194), higher BBB permeability than morphine; transfer half-life; 18–38 min (191)

Methadone	Medium (172)	30 min for onset of action, 1–5 h (132, 185)	4–6 h (132) or longer (195)	None (196)	N/A	41–99% bioavailability (195), 40% permeability (186)

Morphine	High (136, 179)	15–60 min (125, 139)	1.5–4.5 h (IV and IM) (121, 137)	Active: morphine-6-glucuronide (M6G) and hydromorphone. Inactive: morphine-3-glucuronide (M3G) (182).	M6G: 2 h (197); hydromorphone: 5 h (191); M3G: 1.5 h (198)	30% bioavailability, low BBB permeability; transfer half-life; 1.6–4.8 h (191)

Oxycodone	Very high (greater than morphine and hydrocodone) (136, 179)	10–30 min for onset of action (199), peak plasma levels occur ~1 h (137)	2–3 h (199), 3–5 h plasma after oral (137)	Noroxycodone (low activity) and oxymorphone (potency > morphine) (141), both metabolize into noroxymorphone (8–30× morphine’s activity, BBB impermeable) (141)	Noroxycodone is converted slowly into noroxymorphone (200), oxymorphone (7–8 h) (141), noroxymorphone significantly longer than oxycodone (3–5 h but limited BBB permeability) (201)	60–90% bioavailability (142), active transport across the BBB and can reach 3× higher levels in the brain than blood (140, 202)

Remifentanil	Medium, possibly due to low availability (few cases) (203, 204)	1–2 min (143)	3–4 min (IV) (143)	Remifentanil acid, relatively inactive (205)	Negligible (205)	50% bioavailability and BBB equilibration half-life is 2–5 min (205)

Tramadol (e.g., Ultram)	Medium (179, 206);	2–3 h (149, 187)	5.1 h (149)	*O*-desmethyltramadol (M1), an MOR agonist (149)	9 h (149)	Actively transported (207)

The positive correlation between BBB permeability/transport with abuse liability is the cornerstone of the strategically designed novel mu-opioid agonist, NKTR-181, which is analgesic but has limited abuse liability in humans (208, 209). This compound has a poly-ethylene glycol side chain and shows delayed transfer across the BBB (208). It is currently in Phase III clinical trials to treat chronic lower back pain or non-cancer pain (NCT02367820).

### Mixed Opioid Agonists

Another interesting development is the use of ligands that simultaneously bind to and activate multiple receptors to relieve pain. Careful design of these bivalent ligands and their linkers has been shown to increase signaling efficacy of the target receptors, allowing a lower dose of the ligand to be used to achieve the same analgesic effect. Such bitopicity, or action at two sites, was first described for biphalin, a dual enkephalin analog that showed greater analgesic efficacy than enkephalin alone (210). Furthermore, incorporating the pharmacological properties of an opioid that has a reduced abuse liability, i.e., a slow onset of action, a long half-life and low BBB permeability, would result in an effective analgesic that is not rewarding. Several such mixed ligands have now been generated that are based on the structure of buprenorphine (211–213), a partial MOR agonist, kappa opioid receptor (KOR)antagonist and nociceptin receptor ligand (214, 215) with reduced reward liability (172, 173). There are also other bivalent compounds that activate MOR and delta opioid receptors (DOR) (216), MOR and mGluR5 (217), and MOR, DOR and KORs (218).

In summary, we propose that the preclinical examination of novel opioid agonists that are pharmacologically designed to be (1) biased and so able to influence one signaling pathway over another, or (2) show a pharmacological profile that reduces a central duration of action, or (3) are able to signal selectively through mixed receptors, may provide better insight into and predictability of their abuse and lethality profiles. Such novel agonists may also incorporate aspects of each of these designs to obtain the desired clinical outcome. An example of this multi-faceted approach is the family of mixed ligands that are based on the structure of buprenorphine, which may target multiple receptors to enhance analgesia but have a buprenorphine-like pharmacological profile of reduced reward and overdose liability. The specificity and effects of these novel pharmaceutical compounds may be further influenced by the use of a positive allosteric modulator for which a conserved site has been found on MORs, DORs, and KORs (219).

## Treating Pain with Perspective and with the Purpose of Reducing Harm During the Opioid Epidemic

Many pain patients have now found themselves physically and psychologically dependent on their opioid prescriptions, as both fail to relieve their pain in the chronic setting but are also now known to be addictive and harmful with long-term use. We have described the etiology of the opioid epidemic from the financial motivation for the over-prescription of these drugs, to the socioeconomic and physical issues that contribute to pain and addiction-prone populations worldwide. Navigating through the devastation caused by the opioid epidemic requires some perspective. While acknowledging that many opioids are harmful and addictive, they are still the most efficacious class of drugs for analgesia. Here, we aim to guide the refinement of prescription opioid compounds by improving upon the currently available abuse-deterrent formulations. These treatments should maximize analgesic properties by directing ligand bias toward signaling through G-proteins rather than β-arrestins, delaying or minimizing the BBB entry of drugs, minimizing metabolites with pro-addictive or off-target properties and using mixed agonists to provide more specific clinical effects. These strategies have led to the development of some promising compounds that may provide pain relief while minimizing the likelihood of addiction and misuse. Of course, these pharmaceutical agents should only be used following a comprehensive screening strategy to both exclude patients likely to misuse their medications and to identify those who may respond to alternate, non-opioid-based pain-management strategies.

## Author Contributions

This review is the result of a collaboration between basic research scientists and medical professionals. ALS and WW contributed to the conceptualization of the manuscript; edited and wrote the sections of the manuscript concerning the context of the opioid epidemic, pain, biased agonism, and pharmacokinetics. ALS and KH wrote the sections of the manuscript concerning medical interventions, psychiatric comorbidities to pain and addiction, and the epidemiology. ALS, WW, and JH produced the tables of drug properties. OC and GS contributed to writing and editing of the manuscript. All the authors contributed to the final editing and revision of the paper.

## Conflict of Interest Statement

ALS and WW are in communication with Nektar Therapeutics. However, Nektar Therapeutics has had no input in this review. The reviewer KL declared a shared affiliation, with no collaboration, with one of the authors, AS, to the handling Editor.
